# Work Content and Serious Mental Illness among Middle-Aged Men: Results from a 6-Year Longitudinal Study in Japan

**DOI:** 10.1371/journal.pone.0131203

**Published:** 2015-06-29

**Authors:** Hisashi Eguchi, Koji Wada, Yoshiyuki Higuchi, Daisuke Yoneoka, Derek R. Smith

**Affiliations:** 1 Department of Public Health, Kitasato University School of Medicine, Kanagawa, Japan; 2 International Health Cooperation, National Center for Global Health and Medicine, Tokyo, Japan; 3 Department of Health and Physical Education, Fukuoka University of Education, Fukuoka, Japan; 4 Department of Statistical Science, School of Advanced Sciences, the Graduate University for Advanced Studies, Kanagawa, Japan; 5 School of Health Sciences, Faculty of Health and Medicine, University of Newcastle, Ourimbah, New South Wales, Australia; Hamamatsu University School of Medicine, JAPAN

## Abstract

**Objective:**

The present study aimed to determine prospective associations between work content after a working life of more than 20 years and serious mental illness among Japanese men aged 50 to 59 years, using a nationwide population-based survey.

**Methods:**

Data were extracted from a national longitudinal survey of middle-aged and elderly persons previously conducted by the Ministry of Health, Labour and Welfare in Japan. We analyzed data across 10 work content categories for Japanese men who had been working in the same job type or industry for over 20 years. As part pf the survey, participants completed the Kessler (K)6 scale each year to determine their level of psychological distress (with scores ≥13 indicating serious mental illness). Cox discrete time proposal hazard regression analysis was used to examine potential associations between work content and serious mental illness from 2005 to 2010. Further adjustments were made for other sociodemographic characteristics and lifestyle factors.

**Results:**

The current study involved a total of 11,942 participants with a mean (± standard deviation [SD]) of follow-up was 3.4 (± 2.0) years, during which time 892 participants (7.5%) had been classified as having a new-onset serious mental illness. Men who had worked in service jobs and in manufacturing jobs at baseline were more likely to develop serious mental illness than those in managerial jobs (hazard ratio 1.37, 1.30, 95% confidence intervals 1.04–1.80, 1.02–1.65) after adjustment for confounding variables.

**Conclusion:**

These findings suggest that Japanese men aged 50 to 59 years who have worked in service and manufacturing jobs after a working life of over 20 years have an increased risk of serious mental illness during follow-up. Identifying the most at-risk work content category after a working life of over 20 years would be an essential part of providing more effective interventions for psychological distress among Japanese men in this age group.

## Introduction

Lifetime employment in Japan has had a generally positive effect on health status due to increased job security and might have also helped improve the life expectancy of Japanese men following the Second World War [1], [2]. On the other hand, however, recent studies have shown that the age-adjusted mortality rate for the working generation of Japan has changed dramatically, with increased mortality for managers and professionals after the year 2000 [3]. Corporate downsizing following economic recession in the 1990s not only created unemployment, but also increased job demand among remaining employees [4]. These changes in working conditions may have affected employees’ mental health in the long term.

The suicide rate among Japanese men, for example, rose drastically from 26.0 per 100,000 in 1997 to 36.5 per 100,000 in 1998, with major increases observed among men in their 50s [5]. The increase in the suicide rate of middle-aged Japanese men is largely attributed to rapid changes in the industrial structure and working environments following the economic recession in the 1990s [6], [7]. A report from the Japanese Ministry of Health, Labour and Welfare (MHLW) on vital statistics indicated that occupational differences were related to suicide deaths, at least in the year 2000 [8]. A study of company employees in Japan reported a greater prevalence of high job strain among working-class employees (such as manufacturing workers) [9], although another study with a nationally representative sample reported no clear association between occupation and mental health [10]. A previous study of civil servants found that high job strain was less prevalent among those in manufacturing roles [11]. A number of studies in Europe indicated that employees with working-class occupations demonstrate lower reward (or higher effort-reward imbalance) [12–14]. Effort-reward imbalance has been identified by some cross-sectional European studies as being a major risk factor for common mental health disorders such as depression [15–17]. Work content, after many years of working life, may therefore be considered an important sociodemographic factor which may affect mental health.

Given these considerations, identifying the most at-risk work content category after a working life of over 20 years would clearly be an ideal start point for providing more effective intervention for psychological distress among Japanese men in what appears to be a critical age range that being 50–59 years. The present study therefore aimed to investigate the association between work content and serious mental illness during the 5 year period from 2005 to 2010 among men aged 50 to 59 years who had worked in the same job type or industry for more than 20 years at baseline.

## Methods

The present study utilized data from the Longitudinal Survey of Middle-aged and Elderly Persons (LSMEP), an ongoing nationally representative cohort study conducted by the Japanese MHLW [18]. The LSMEP aimed to investigate the health conditions, work situations and participation in social activities among middle-aged and elderly people by collecting data continuously from a nationally representative sample of men and women who were aged between 50 and 59 years at the end of October, 2005. The LSMEP also sought to inform policy development based on the results.

The first wave of data collection began in November 2005, and subsequent waves were conducted on a yearly basis. Participants in the first wave were recruited nationwide in November 2005, through a two-stage random sampling procedure. First, 2,515 districts were randomly selected from the 5,280 districts used by the MHLW nationwide, population-based Comprehensive Survey of the Living Conditions of People on Health and Welfare, conducted in 2004. These 5,280 districts had been randomly selected from about 940,000 national census districts. Following this, 40,877 residents aged 50–59 years (as at October 30, 2005) were randomly selected from the selected districts, according to population size. All LSMEP waves used a self-administered questionnaire conducted by mail and included the same questions about work content and psychological distress. The survey also gathered information on participants’ family situation, health status and work status on an annual basis to help inform policy decisions for older people. The 2005 questionnaire were physically distributed to participants’ homes, where they were complete by participants as at November 2, 2005, and physically collected several days later. A total of 34,240 individuals responded (response rate: 83.8%). The second to sixth survey waves were conducted from 2006 to 2010 and consisted of 32,285; 30,730; 29,605; 28,736 and 26,220 respondents, respectively (with response rate of between 91.8% and 97.3%). For these surveys, the questionnaire was mailed only to individuals who had participated in either of the previous two waves, and had returned a completed questionnaire. No new respondents were added after first wave. Participants in the sixth survey were therefore aged between 55 and 64 years when that survey conducted.

Ethical approval for the nationwide population-based survey was obtained by the MHLW. The corresponding author (KW) obtained permission to use individual data from the 2005 to 2010 LSMEP questionnaire for purposes other than those originally intended by the MHLW. As the current study comprised retrospective analyses of national surveillance data that were free of any personally identifiable information, ethics approval was not required for the present study.

### Study Population

Participants who met the following criteria from baseline were excluded from the present study: those who did not work in the same job type or industry for more than 20 years (N = 1,588); those with existing, serious mental illness (n = 1,923); those missing covariate data (n = 5,544), and those with missing data regarding prevalence of serious mental illness (n = 1,357). The remaining 11,942 male employees without serious mental illness at baseline constituted the study population.

### Outcome (Serious mental illness)

The questionnaire included the K6 scale originally developed by Kessler et al. to detect general psychological distress [19]. The Kessler (K)6 scale is reported to effectively detect major depression and dysthymia according to established DSM-IV criteria. It consists of six items and measures the extent of psychological distress using a five-point response option from 0 (none of the time) to 4 (all of the time). Total scores range from 0–24, with higher scores indicating proportionally greater psychological distress. The K6 scale has since been translated into Japanese and has been shown to have acceptable internal consistency, reliability and validity [20]. According to the recommended K6 cutoff point, participants with a total score of 13 or more (13–24) were defied as having serious mental illness, while a score of 0–12 suggests no mental illness [19], [21], [22]. The prevalence of serious mental illness at baseline, and numbers of participants identified as new-onset serious mental illness at one of the five follow-ups (subsequent survey waves), were recorded.

### Predictor (Work content)

Participants were asked to select their work content from one of the following categories on the baseline questionnaire: 1) professional or technical job; 2) managerial job; 3) clerical job; 4) sales job; 5) service job; 6) security / protection job; 7) agricultural / forest/ fishery job; 8) transport / communication job; 9) production, construction or craft job; and 10) other unclassified job. This classification was based on the International Standard Classification of Occupations [23]. Participants were also asked about years of service in their selected job type or industry.

### Other Covariates

The demographic and lifestyle characteristics measured were based on previous studies which looked at risk factors for mental health issues, and included age, marital status, employment contract [24], smoking status [25], frequency of alcohol drinking [26], frequency of physical exercise [27], diabetes [28], heart disease [29], stroke [30], hypertension [31], hyperlipidemia [32] and cancer [33] at baseline, as well as educational attainment [34] at the second wave. Age was evaluated as a continuous variable. Marital status was classified into three groups (currently married, divorced / widowed and never married). Education attainment in the second wave was classified into four groups (junior high school / high school, college / vocational school, university and over, other). Smoking status was classified into three groups (current smoker, ex-smoker and lifetime nonsmoker). Frequency of alcohol drinking was classified into four groups (more than 5 times per week, more than once per month, not at all, and unknown). Frequency of physical exercise was classified as either more than once a month or others. Employment contract was assessed by a question on employment status with nine response options. Employment contract was classified into four groups: regular, non-regular (part-time employee, dispatched employee from temporary labor agency, or contract / entrusted employee), self-employed, and other (occupations that were not classifiable). Past history or currently medicated chronic physical conditions were assessed by a multiple choice questionnaire and diabetes, heart disease, stroke, hypertension, hyperlipidemia and cancer were chosen as covariates.

### Statistical Analysis

Potential associations between work content at baseline after a working life of more than 20 years and the chosen variables at baseline were examined using chi square tests for discrete variables and analysis of variance for continuous variables. Statistical analyses were based on prevalence rates of total serious mental illness during the 5-year follow-up period (2005–2010). After the associations between work content and the study variables at baseline were examined, a survival analysis was conducted to test the effectiveness of the association for the time to onset of serious mental illness, while controlling for censoring effects due to the length of follow-up or the completion of follow-up without the onset of serious mental illness. Length of follow-up for each participant was represented by the number of years between the baseline and the onset of serious mental illness, loss to follow-up or the end the 6-year follow-up period, whichever came first. Cox’s discrete time proportional hazard regression analysis was used to examine the association between work content and the prevalence of serious mental illness, including time-to-event information [35]. Hazard ratios (HR) were estimated first after adjusting for age, and then after adjusting for age, marital status, employment contract, smoking status, frequency of drinking alcohol, frequency of physical exercise, diabetes, heart disease, stroke, hypertension, hyperlipidemia and cancer at baseline, and educational attainment at the second wave. In addition, Kaplan-Meier curves and log-rank tests were used to estimate the cumulative prevalence of covariates which indicated a significant HR. The estimated HRs of serious mental illness and 95% confidence intervals (CI) were calculated taking managerial jobs as the reference group, similar to previous study [9].

## Results

The current study involved a total of 11,942 participants with a mean (± SD) follow-up period of 3.4 (± 2.0) years. Of the participants, 892 (7.5%) had developed serious mental illness during the follow-up period, with a mean (± SD) follow-up period of 2.6 (± 1.3) years. The mean (± SD) follow-up period for participants who did not develop serious mental illness was 3.5 (± 2.0) years.

Table 1 indicates the association between work content and the examined variables at baseline. Participants with managerial, service, security and manufacturing jobs tended to be older, while those in clerical jobs were younger. More participants in manufacturing jobs were unmarried than those in managerial jobs. Work content was not associated with educational attainment. More participants in transportation or communication jobs were current smokers than those in clerical jobs. Participants in professional or technical jobs exercised more frequently than those in agricultural, forestry or fishery jobs. Alcohol drinking was reported more frequently by participants with managerial jobs than those with security jobs. The likelihood of regular employment was higher in participants in professional or technical jobs than in those with agricultural, forestry or fishery jobs.

**Table 1:** Baseline association between contents of the work and the studied variables (n = 11,942).

	Managerial job	Professional and technical job	Clerical job	Sales job	Service job	Security job	Agricultural forestry and fishery job	Transportation and communications job	Manufacturing job	Others	p value^a^
No. of subjects	2,033	3,267	878	1026	933	266	438	786	1,677	638	….
Mean age, y	54.9	54.5	54.4	54.8	54.9	55.0	55.0	54.9	54.9	54.9	<0.001
Marital status, %											
Married	94.8	90.4	92.5	91.5	88.2	86.5	89.0	87.3	85.0	84.2	<0.001
Divorced/widowed	3.0	4.3	3.6	4.1	5.7	7.1	4.3	6.9	5.8	6.6	
Never married	2.2	5.4	3.9	4.4	6.1	6.4	6.6	5.9	9.2	9.2	
Education, %											
Junior high school/high school	65.9	65.4	65.3	64.4	66.6	61.3	66.4	65.4	63.2	65.8	0.419
College/vocational school	7.4	7.2	9.9	8.5	9.6	9.4	7.3	9.0	7.8	7.5	
Universtiy and over	26.0	26.5	23.9	26.4	23.3	28.9	25.8	25.1	28.2	26	
Others	0.7	0.9	0.9	0.7	0.5	0.4	0.5	0.5	0.8	0.6	
Employment contract, %											
Regular	91.9	64.7	94.5	61.1	54.8	77.1	12.6	79.4	75.1	44.5	<0.001
Non-regular	2.4	4.0	4.0	4.5	12.3	19.5	3.7	9.4	11.2	16.0	
Self employed	5.2	30.0	1.3	34.2	32.3	1.9	81.7	10.7	12.5	27.7	
Others	0.5	1.3	0.2	0.2	0.6	1.5	2.1	0.5	1.2	11.8	

a Test for heterogeneity.

Table 2 shows the prediction for new-onset serious mental illness during the follow-up period. Participants with service jobs displayed an increased risk for serious mental illness compared with participants in managerial jobs, after adjusting for age (HR: 1.44, 95%CI: 1.10–1.89). The result was same after adjusting for confounding factors (HR: 1.37, 95%CI: 1.04–1.80). Similarly, participants in manufacturing jobs displayed an increased risk for serious mental illness when compared with participants in managerial jobs, after adjusting for age (HR: 1.34, 95%CI: 1.06–1.69), and after adjusting for confounding factors (HR: 1.30, 95%CI: 1.02–1.65).

**Table 2:** Hazard ratios for incidence of serious psychological distress associated with content of the work and experience of unemployment among male participants (N = 11,942).

Occupation	Cases / non-cases		Crude model		Age adjusted model		Fully adjusted model ^a^	
	N	(%)	HR	95%CI	HR	95%CI	HR	95%CI
Occupation								
Managerial job	138 / 1895	(7.3)	1.00		1.00		1.00	
Professional and technical job	228 / 3039	(7.5)	1.06	0.86–1.31	1.05	0.85–1.30	1.00	0.81–1.24
Service job	84 / 849	(9.9)	1.44	1.10–1.89	1.44	1.10–1.89	1.37	1.04–1.80
Manufacturing job	143 / 1534	(9.3)	1.34	1.01–1.69	1.34	1.06–1.69	1.30	1.02–1.65
Sales job	87 / 939	(9.3)	1.30	1.00–1.70	1.30	0.99–1.70	1.23	0.94–1.62
Clerical job	60 / 818	(7.3)	1.00	0.74–1.36	0.99	0.73–1.34	1.00	0.74–1.36
Transportation and communications job	52 / 734	(7.1)	1.00	0.73–1.38	1.00	0.73–1.38	0.96	0.70–1.33
Agriculture, forestry and fishery job	36 / 402	(9.0)	1.26	0.87–1.81	1.26	0.87–1.82	1.11	0.75–1.64
Security job	16 / 250	(6.4)	0.86	0.51–1.49	0.86	0.52–1.45	0.87	0.52–1.46
Others	48 / 590	(8.1)	1.23	0.89–1.71	1.23	0.88–1.71	1.15	0.82–1.61
Marital status								
Married	795 / 9927	(7.4)	1.00		1.00		1.00	
Divorced/widowed	54 / 505	(9.7)	1.33	1.01–1.75	1.34	1.01–1.76	1.31	0.99–1.72
Never married	43 / 618	(6.5)	0.94	0.69–1.28	0.93	0.96–1.01	0.91	0.67–1.24
Education							1.00	
Junior high school/high school	574 / 7205	(7.4)	1.00		1.00		0.97	0.76–1.24
College/vocational school	71 / 886	(7.4)	0.96	0.75–1.23	0.96	0.75–1.23	1.03	0.89–1.20
University and over	238 / 2881	(7.6)	1.04	0.89–1.21	1.04	0.86–1.21	1.43	0.74–2.77
Others	9 / 78	(10.3)	1.45	0.75–2.79	1.43	0.74–2.77	0.92	0.85–1.00
Employment contract								
Regular	596 / 7781	(7.1)	1.00		1.00		1.00	
Non-regular	77 / 731	(9.5)	1.44	1.13–1.82	1.46	1.15–1.85	1.39	1.09–1.77
Self employed	206 / 2376	(8.0)	1.18	1.01–1.39	1.19	1.02–1.40	1.17	1.00–1.38
Others	13 / 162	(7.4)	1.14	0.66–1.97	1.15	0.66–1.98	1.10	0.63–1.91

a Adjusted for age, marital status, smoking, frequency of drinking alcohol, frequency of exercise, diabetes, heart disease, stroke, hypertention, hyperlipidemia, cancer, employment contract at baseline and education at 2nd wave.

No significant association was found between educational attainment and serious mental illness. For employment contract, those with non-regular employment (HR: 1.39; 95%CI: 1.09–1.77) and who were self-employed (HR: 1.17; 95%CI: 1.00–1.38) were shown to be at higher risk of serious mental illness when compared with regular employees.

**Fig 1:**
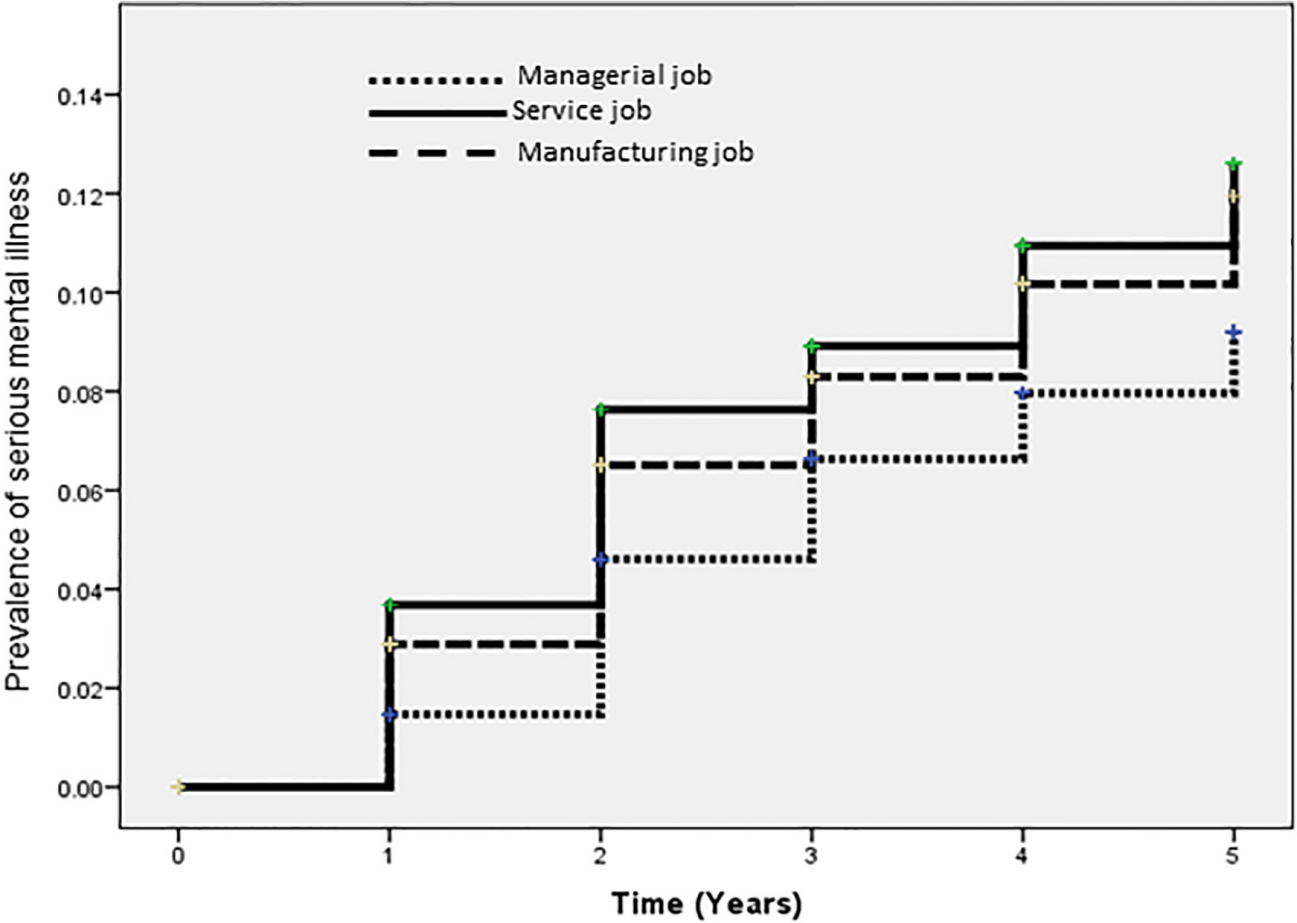
Kaplan–Meier curves and log-rank tests of the prevalence of serious mental illness. Kaplan–Meier curves indicate follow-up periods (in years) and cumulative prevalence rate of serious mental illness (p = 0.007).

The cumulative prevalence of serious mental illness was analyzed by the Kaplan-Meier method using three work content categories (managerial jobs, service jobs and manufacturing jobs), and revealed significant differences in work content (p<0.01; log-rank test) (Fig 1).

## Discussion

Our study investigated the potential associations between work content after a working life of over 20 years and serious mental illness in Japanese men aged 50–59 years. The main findings suggest that work content is associated with serious mental illness, at least among this particular cohort. After adjusting for sociodemographic factors, we found that workers in service and manufacturing jobs were greater risk of serious mental illness than those in managerial jobs. Even though employees in this cohort might have experienced unemployment or a change in their work content, our findings suggest that health promotion authorities should carefully consider work content when implementing interventions to reduce serious mental illness among 50 to 59 year-old men in Japan.

Workers in service jobs had the greatest risk of serious mental illness in the current study. In Japan, a special report of vital statistics by the MHLW found that workers in service jobs had the second highest suicide mortality after workers employed in agricultural / forestry / fishery jobs in the year 2000 [8]. Many studies have suggested that the high emotional demands and specific characteristics of service jobs and other caring and teaching jobs are risk factors for common metal disorders [15], [36], [37]. In service industries in Japan, labor productivity and the provision of holiday pay are low [38], [39]; while employee turnover and the incidence of occupational injury, are high. The relatively poor work environment and job characteristics of service jobs in Japan (compared with their European counterparts) may have a harmful effect on employees' mental health.

Our findings indicated that Japanese men in their 50–59 years and working in manufacturing jobs, after a working life of over 20 years, had a higher risk of serious mental illness when compared to workers in managerial jobs. In Japan, increased susceptibility to mental illness in workers in low occupational classes has been attributed to adverse psychosocial work environments [9], [40], [41]. It is also noteworthy that since the economic recession of the 1990s, international competition, a corresponding rise in job insecurity and a more competitive working climate has also affected male working in manufacturing jobs [42]. The economic recession appears to have had a strong effect on the increase in suicides, especially in middle-aged men in lower occupational classes [43], [44].

Although a particular strength of our current study is no doubt the large population-based longitudinal design, some limitations should still be considered when interpreting the findings. First, unmeasured factors may have affected the association between work content and the prevalence of serious mental illness; although it is worth noting that a range of possible confounding factors were considered in our analysis. A possible confounding variable that was not specifically considered was household income, and this variable may be worth investigating in future research. Second, there may have been some measurement error in the work content categorization. For example, if participants with managerial jobs covered a number of different sectors such as sales or service jobs, misclassification might have occurred in their answers; meaning the association between work content and serious mental illness may be weak. Third, working situation and baseline variables such as marital status and lifestyle behaviors might have changed over time, which may have affected some associations. For example, participants with a history of unemployment and changing work content during the follow-up period, but without the experience of unemployment at baseline, could have been included in our sample. In addition, working situation and baseline variables such as marital status and lifestyle behaviors might have changed over time, which may also have affected some statistical associations. Fourth, unpredictable work content misclassification may lead to bias. Such measurement errors are probably undifferentiated and could lead to underestimations of the differences. Fifth, females were excluded from the analyses. In a previous study, the association between occupation and mental health was found to differ by sex [45], [37]. Therefore, future studies should consider investigating the potential associations between work content and mental health in Japanese women. Finally, the questionnaire was answered during the same time each year (once a year). This may lead to overestimation of the effect of work content.

Mental health issues clearly represent a major public health issue in contemporary Japanese society [46] and the present study set out to investigate associations between work content and serious mental illness in middle-aged Japanese men. Identifying the most at-risk work content after a working life of over 20 years represents an essential step in providing the most effective interventions for serious mental illness targeted at workers in specific work contents in Japan, as other countries.

## Conclusion

Overall, the findings from the current study suggest that Japanese men aged 50–59 years who work in service and manufacturing jobs, and who have worked in that job type or industry for over 20 years, have an increased risk of serious mental illness. Identifying the most at-risk work content category after a working life of over 20 years would therefore comprise an essential part of more effective interventions for psychological distress among Japanese men in this particular age group.
